# Effects of Iron Overload on the Activity of Na,K-ATPase and Lipid Profile of the Human Erythrocyte Membrane

**DOI:** 10.1371/journal.pone.0132852

**Published:** 2015-07-21

**Authors:** Leilismara Sousa, Israel J. P. Garcia, Tamara G. F. Costa, Lilian N. D. Silva, Cristiane O. Renó, Eneida S. Oliveira, Cristiane Q. Tilelli, Luciana L. Santos, Vanessa F. Cortes, Herica L. Santos, Leandro A. Barbosa

**Affiliations:** 1 Laboratório de Bioquímica Celular, Universidade Federal de São João del Rei, Campus Centro-Oeste Dona Lindú, Av Sebastião Gonçalves Coelho 400, 35501–296, Divinópolis, Brazil; 2 Laboratório de Estudos em Neurociências das Epilepsias e Comorbidades, Universidade Federal de São João del Rei, Campus Centro-Oeste Dona Lindú, Av Sebastião Gonçalves Coelho 400, 35501–296, Divinópolis, Brazil; 3 Laboratório de Biologia Molecular, Universidade Federal de São João del Rei, Campus Centro-Oeste Dona Lindú, Av Sebastião Gonçalves Coelho 400, 35501–296, Divinópolis, Brazil; CINVESTAV-IPN, MEXICO

## Abstract

Iron is an essential chemical element for human life. However, in some pathological conditions, such as hereditary hemochromatosis type 1 (HH1), iron overload induces the production of reactive oxygen species that may lead to lipid peroxidation and a change in the plasma-membrane lipid profile. In this study, we investigated whether iron overload interferes with the Na,K-ATPase activity of the plasma membrane by studying erythrocytes that were obtained from the whole blood of patients suffering from iron overload. Additionally, we treated erythrocytes of normal subjects with 0.8 mM H_2_O_2_ and 1 μM FeCl_3_ for 24 h. We then analyzed the lipid profile, lipid peroxidation and Na,K-ATPase activity of plasma membranes derived from these cells. Iron overload was more frequent in men (87.5%) than in women and was associated with an increase (446%) in lipid peroxidation, as indicated by the amount of the thiobarbituric acid reactive substances (TBARS) and an increase (327%) in the Na,K-ATPase activity in the plasma membrane of erythrocytes. Erythrocytes treated with 1 μM FeCl_3_ for 24 h showed an increase (132%) in the Na,K-ATPase activity but no change in the TBARS levels. Iron treatment also decreased the cholesterol and phospholipid content of the erythrocyte membranes and similar decreases were observed in iron overload patients. In contrast, erythrocytes treated with 0.8 mM H_2_O_2_ for 24 h showed no change in the measured parameters. These results indicate that erythrocytes from patients with iron overload exhibit higher Na,K-ATPase activity compared with normal subjects and that this effect is specifically associated with altered iron levels.

## Introduction

Iron is an essential chemical element for human life because it participates in fundamental physiological processes, such as oxygen transport, mitochondrial respiration, DNA synthesis, oxidative energy production and the inactivation of free radicals. The average amount of serum iron in the normal adult body varies from 65–176 μg/dL[1–2], with feeding [3] and erythrocyte senescence [1, 4]representing the major sources.

Various enzymes and cell types are involved in iron metabolism, including enterocytes, erythrocytes, macrophages and hepatocytes [1]. Changes in protein expression can severely impact iron homeostasis; for example, decreased synthesis of the hormone hepcidin, an inhibitor of the iron transport protein ferroportin, can lead to a progressive and pathological accumulation of iron in the body [5], especially in parenchymal organs [6]. An accurate diagnosis requires the detection of increases in the main biochemical markers of iron overload, i.e., serum ferritin and the transferrin saturation index; the appearance of clinical manifestations should be noted, and mutations should be identified by genetic testing [7]. Iron overload is known to cause cellular damage because of its multiple important functions, such as the mediation of lipid peroxidation caused by reactive oxygen species (ROS) [6]. Additionally, lipoperoxidation changes the lipid composition, fluidity and permeability of the plasma membrane, which may in turn affect the function of resident enzymes such as the Na,K-ATPase[8–9].

Ionic transport is essential for the maintenance of viable erythrocytes in blood. Several pathways mediate the water and solute balance in erythrocytes, and cellular volume is regulated by the monovalent cation concentration[10]. The transport of anions and cations through the membrane is regulated by several enzymes, including the Ca^2+^-ATPase, Na^1+^/Ca^2+^ exchanger, Na-K-Cl cotransporter (NKCC), band-3 protein, H-ATPase and Na,K-ATPase[11–14]. Na,K-ATPase is a key protein that regulates the cellular volume of erythrocytes, which is fundamental for avoiding hemolysis, and has a huge impact on the deformability of erythrocytes, which is necessary to withstand blood pressure and pass through narrow vessels; thus, it is a crucial factor for erythrocyte viability [15]. Moreover, Na,K-ATPase activation increases glycolysis in erythrocytes[16–17].

Na,K-ATPase is a dimeric integral membrane enzyme that belongs to the class of P-type ATPases. Na,K-ATPase catalyzes the transport of 3 sodium ions outside the cells and 2 potassium ions into the cells, thereby generating an asymmetric gradient across the plasma membrane [18]. This gradient is important for the transport of glucose and amino acids through the membrane and for maintaining the cell resting potential [19]. The Na,K-ATPase exists as several isoenzymes, which result from the variable combinations of molecular forms of the α and β subunits. A γ subunit is also encoded by the FXYD2 gene and forms part of the Na,K-ATPase in some tissues[20–21]. The α subunit contains the catalytic domain and has a molecular weight between 110 and 112 kDa, 10 transmembrane segments and a small ectodomain. The α subunit contains binding sites for sodium and potassium ions, ATP and cardiotonic steroids such as ouabain, which is an inhibitor of the enzyme [22]. The β subunit is a glycoprotein with a molecular weight between 40 and 60 kDa. The β subunit contains a regulatory domain in its transmembrane helix, which contains a glycosylation site that is essential for the normal activity of the enzyme [23]. The γ subunit has a single transmembrane domain, an approximate molecular weight of 7 kDa, and a hydrophobic character that facilitates its role in the modulation of Na,K-ATPase activity[24–25]. These subunits may also appear as different isoforms. The subunit isoforms that are found in erythrocytes are α1, α3, β1, β2, β3 and γ [26].

The activity of Na,K-ATPase is modulated by the intracellular and extracellular ATP and sodium concentrations. The affinity of Na,K-ATPase for sodium and potassium seems to be modulated by tissue-specific factors, such as the lipid composition of the membrane[20, 27–28]. It has been shown that the activity of Na,K-ATPase is elevated in renal epithelial cells of Madin-Darby canines (MDCK), which leads to an increase in iron transport and low concentrations of potassium [29]. Because Na,K-ATPase is important in the regulation of several properties in erythrocytes, it has been hypothesized that this enzyme may be used as a marker for characterizing the cell-damage status due to iron overload.

In the current work, we report the biochemical investigations of the effects of iron overload on Na,K-ATPase activity and lipid peroxidation in human erythrocytes.

## Materials and Methods

### 2.1. Patients

Blood samples were obtained from patients experiencing iron overload with ferritin values greater than 200 μg for women and 300 μg for men. Patients with metabolic syndrome, alcohol utilization, chronic inflammation or hepatic disease were excluded from this study. In this context, the patients who were included could be classified as having hemochromatosis according to the American Hemochromatosis Guidelines; however, in Brazil, these patients do not have a confirmed genetic diagnosis due to a lack of financial resources.

The patients in this study were under standard care, which includes therapeutic phlebotomy due to high iron and ferritin concentrations. The samples were collected at the outpatient clinic of the Hemonúcleo Regional Divinopolis / MG, Brazil, an outpatient unit that belongs to the Hemominas Foundation and is used for conducting therapeutic phlebotomy. Blood samples from whole blood bags with CPDA-1 (Citrate Phosphate Dextrose Adenine-1) and a tube with EDTA (ethylenediamine tetraacetic acid) were collected from patients treated between September and December 2012. As a control group, we selected healthy volunteers with ferritin values lower than 200 μg for women and 300 μg for men and with similar ages to the patients and no clinical symptoms of iron overload. The information about clinical manifestations was collected through a questionnaire answered by the patients.

This study was approved by the Hemominas Foundation Research Ethics Committee and by the Ethics in Research involving Human Subjects of the Universidade Federal de São João del Rei, Campus Centro-Oeste Dona Lindu—CEPES / CCO, with the number 138.241. All of the procedures followed in this study were in accordance with the ethical standards of the responsible committees on human experimentation (institutional and national) and with the Helsinki Declaration of 1975. All patients were informed about the study and consented to participate by signing an Informed Consent Form.

### 2.2. Genetic analysis

Genomic DNA of patients was extracted from peripheral blood leukocytes and quantified with NanoDrop 2000c spectrophotometer, Thermo Scientific. The Polymerase Chain Reaction (PCR) was performed to amplify regions of exons 2 and 4 of the gene that codifies for the human hemochromatosis protein (HFE). Exon 2 was amplified with the forward primer (E2F, ACATGGTTAAGGCCTGTTGC 5'- 3') and the reverse primer E2R (GCCACATCTGGCTTGAAATT 5'-3'), and exon 4 with the foward primer E4F (TGCCTCCTTTGGTGAAGGTGAC 5'-3') and the reverse primer E4R (CTCAGGCACTCCTCTCAACC 5'-3'). All primers were manufactured by Eurofins MWG Operon. PCR products of exon 2 were digested with the restriction enzymes Bcl I (Jena Bioscience, Jena, Germany), and Hinf I (Fermentas, Vilnius, Lithuania), to detect mutations H63D and S65C, respectively. PCR products of exon 4 were digested with the restriction enzyme Afa I (Invitrogen, Carlsbad, CA, USA). PCR products were analyzed by eletrophoresis in non-denaturing 8% polyacrylamide gel stained with ethidium bromide.

### 2.3. Preparation of erythrocyte membranes

Preparations of cytoplasm-free membrane ghosts were done according to Rega et al. [30] with some modifications. The blood concentrates were spun at 6,500×g for 10 min at 4°C. The precipitates were dissolved in a solution of 20 mM Tris–HCl (pH 7.4), 130 mM KCl, and 0.6 mg/ml phenylmethylsulfonyl fluoride (PMSF). This suspension was spun at 6,500×g for 10 min at 4°C. The cells were submitted to lysis by freezing in liquid nitrogen followed by thawing at room temperature (25°C). The lysates were then mixed with 5 mM 4-2-hydroxyethyl-1-piperazineethanesulfonic acid (HEPES; pH 7.4), 1 mM ethylenediamine tetraacetic acid (EDTA) and 0.6 mg/ml PMSF and spun again at 9,000×g for 10 min at 4°C. This centrifugation step was repeated four times, and the resulting pellet was dissolved in 10 mM HEPES (pH 7.4), 130 mM KCl, 0.5 mM MgCl_2_, and 0.05 mM CaCl_2_, and finally spun at 9,000×*g* for 10 min, mixed with a small volume of this last buffer, and stored in nitrogen until use.

### 2.4. Determination of oxidized iron (Fe^3+^) in plasma samples

The iron ion concentration was estimated from the absorbance of the colored complex formed by reaction with KSCN Fe^3+^ [31]. The absorbance was read at 480 nm and the calibration curve was first established with a 1 mmol / L FeCl_3_ solution.

### 2.5. Determination of thiobarbituric acid reactive substances (TBARS)

The measurement of TBARS was conducted using the methodology described by Buege and Aust [32], with ghost membrane samples obtained as described above.

### 2.6. Determination of Na,K-ATPase activity

The Na,K-ATPase activity was determined at 37°C, by measuring the inorganic phosphate released in the reaction containing HEPES buffer (50 mM HEPES, pH 7.5, 20 mM KCl, 2 mM MgCl2, 120 mM NaCl), ATP 3 mM and ghost membranes (47 mg), in a final volume of 0.25 μl. The experiments were performed in the presence and absence of ouabain, to specifically determine the activity of Na,K-ATPase. The reaction was initiated by the addition of ATP to HEPES buffer and stopped with 100 mL of 1% SDS (w/v) after 60 minutes. The inorganic phosphate produced by the hydrolysis of ATP was determined spectrophotometrically, according to Fiske and Subbarow [33], at 660 nm.

### 2.7. Extraction and quantification of phospholipids and cholesterol in the plasma membrane

The lipid extraction was performed with chloroform:isopropanol (14:11), according to the method of Oaklanderand Rose [34] and Vokurková, Nováková et al [35]. After phase separation, the organic layer was dried and re-suspended in a known volume of chloroform. The total phospholipids were measured by quantification of inorganic phosphate released from hydrolysis, according to the procedure described by Chen and Toribara [36], and the total cholesterol was determined according to the method of Higgins [37].

### 2.8. Statistical analysis

Statistical analysis were performed as appropriate, using the following tests: D'Agostino-Pearson and Shapiro-Wilk normality tests, Student’s t test and Mann Whitney test to compare means, chi-square test and Fisher exact test to compare proportions, for determination of correlation between data, Pearson and Spearman correlation and ANOVA to determination of variance. The alpha set for the analysis of significance was 5.0% and the software used was GraphPad Prism 5.

## Results

### 3.1 Clinical manifestations and genetic analysis

This study included 24 patients suffering from iron overload (21 men and 3 women), with a median age of 49 years. Two of the patients were brothers. Fourteen subjects were recruited for the control group, 10 men and 4 women, who had a median age of 45 years. Each patient was treated an average of 6 times, with a range of 1 to 22 phlebotomies and an average prescribed volume of 427 ml of whole blood withdrawal. The clinical manifestations did not show any correlation to the number or frequency of phlebotomies undergone by patients (p>0.05). The main clinical manifestations reported by patients are listed in Table 1 according to age group.

**Table 1:** Main clinical manifestations reported by patients with hyperferritinemia grouped by age.

Clinical manifestations	Less than 50 years	Older than 50 years	*P* *
Chronic liver disease	7 (58,3%)	4 (33,3%)	0,414
Diabetes	0 (0%)	3 (25%)	-
Arthropathy	5 (41,7%)	8 (66,7%)	0,414
Fatigue/weakness	7 (58,3%)	6 (50%)	1,000
Skin hyperpigmentation	2 (16,7%)	9 (75%)	0,012
Sudoresis	4 (33,3%)	3 (25%)	1,000
Impotence	0 (0%)^†^	2 (18,2%)^‡^	-

* X^2^

^**†**^ n = 10

^**‡**^ n = 11

The frequencies of C282Y and H63D mutations were 35.4% and 14.6%, respectively, in the patient group and 17.9% and 3.6%, respectively, for the control group. The HFE genotype frequency is shown in Table 2. Neither case nor control subjects presented the S65C mutation in the HFE gene or were compound heterozygous for C282Y or H63D. The most frequent genotype for case patients was H63D/WT, with a frequency of 45.8% compared with 35.7% for the control group; however, this difference is not statistically significant.

**Table 2:** Frequency of HFE genotypes in patients and control subjects.

HFE genotype	Patients		Control		
	n	Genotypic frequency (%)	n	Genotypic frequency (%)	*P* *
C282Y/C282Y	3	12,5	0	0,0	-
C282Y/WT^†^	1	4,2	1	7,1	1,0
H63D/H63D	3	12,5	0	0,0	-
H63D/WT	11	45,8	5	35,7	0,491
No mutation	6	25,0	8	57,2	0,081
Total	24	100,0	14	100,0	-

* X^2^

^†^WT (wild type)

### 3.2. Influence of iron overload on the oxidative state of erythrocytes

**Fig 1:**
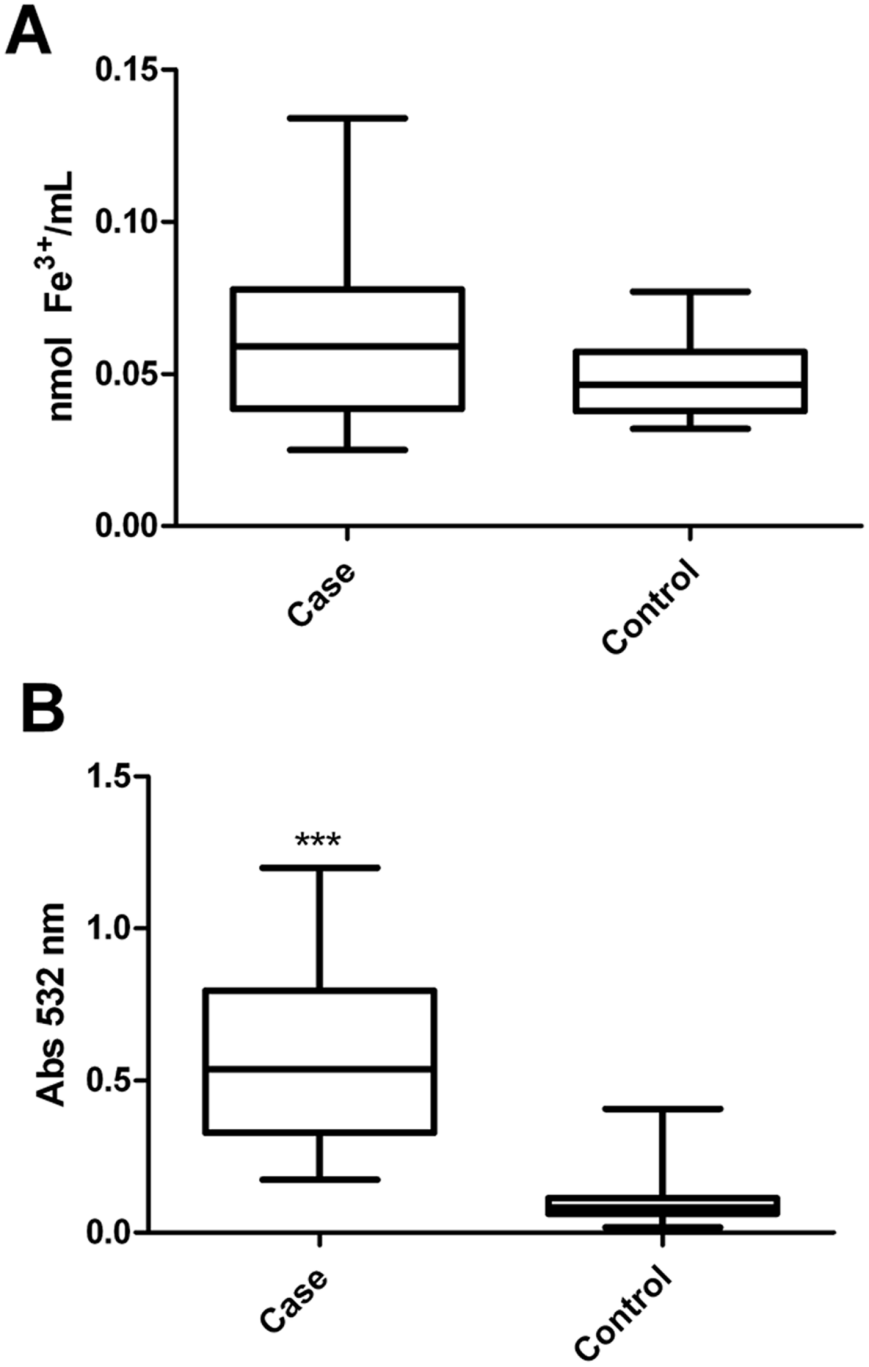
Iron overload effect on oxidative stress. (A) Content of Fe^3+^ in the residual plasma of individuals with or without iron overload: n = 24 (case) and n = 14 (control), p = 0.087 (Mann Whitney). (B) Lipid peroxidation in subjects with or without iron overload: n = 14 (case) and n = 14 (control), p<0.0001 (Student's t test). All experiments were performed in triplicate and the line in bars represented the mean and the error bar denotes SEM.

The concentration of erythrocytes obtained from the patients (0.065 ± 0.029, n = 24) is not significantly different from that of control subjects (0.048 ± 0.014, n = 14; p = 0.087; Fig 1A). Peroxidated lipids, measured as the amount of TBARS, showed an increase of 446% in erythrocyte membranes from the case group (0.442 ± 0.043, n = 24) compared to the control group (0.099 ± 0.014, n = 14; p<0.0001; Fig 1B).

### 3.3. Iron overload changes the lipid profile of erythrocyte plasma membranes

**Fig 2:**
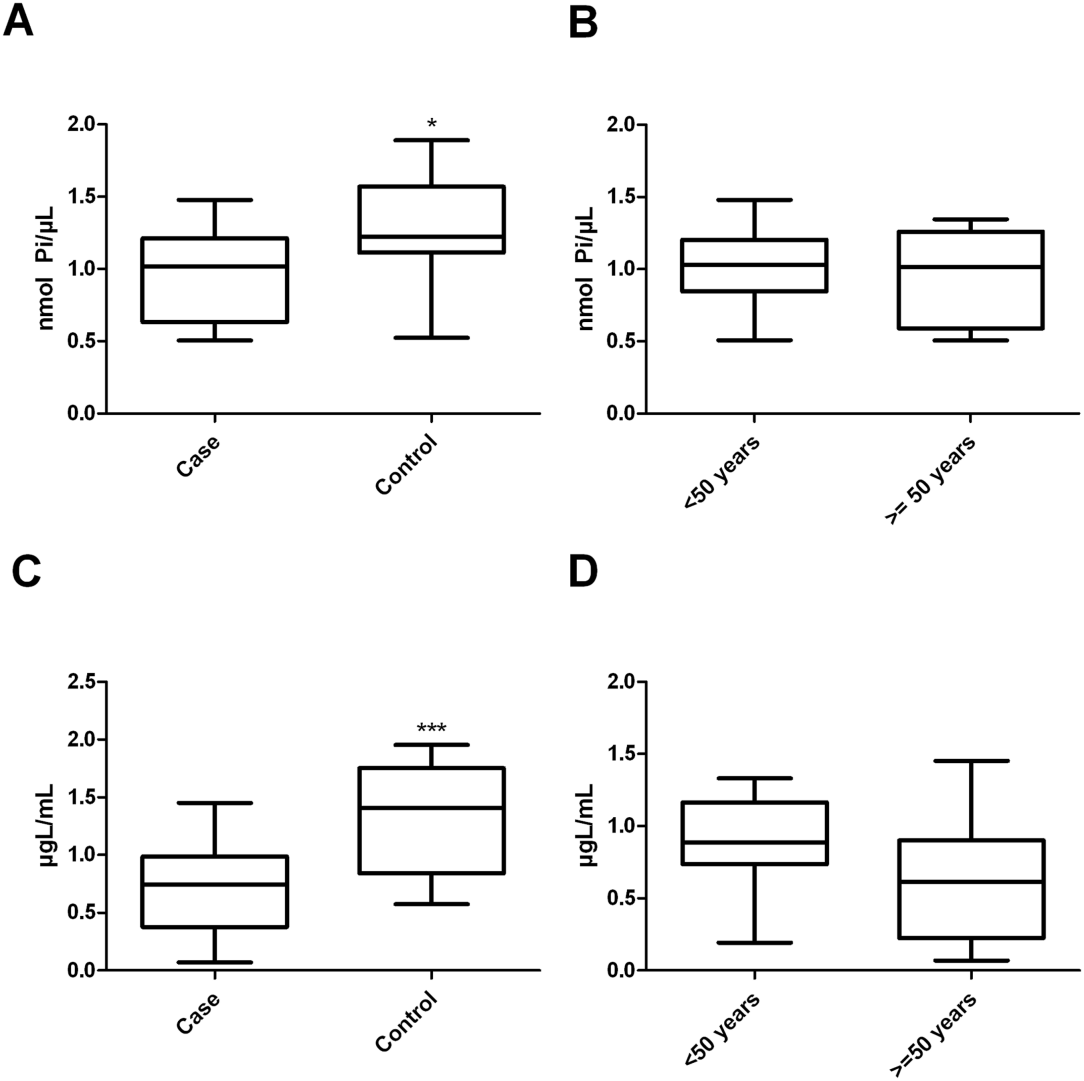
Influence of iron overload on lipid profile of the erythrocyte plasma membrane. (A) Total phospholipids: n = 23 (case) and n = 14 (control), p = 0.013 (Student's t test). (B) Total phospholipids of patients with iron overload, stratified by age: n = 11 (<50 years) and n = 12 (> = 50 years), p = 0.554 (Student's t test). (C) Total membrane cholesterol: n = 23 (case) and n = 14 (control), p<0.001 (Student's t test). (D) Total membrane cholesterol of patients with iron overload, stratified by age: n = 11 (<50 years) and n = 12 (> = 50 years), p = 0.241 (Student's t test). All experiments were performed in triplicate and the line in bars represented the mean and the error bar denotes SEM.

No existing studies demonstrate whether iron overload changes the lipid profile of erythrocyte plasma membranes. These changes may result from the peroxidation of lipids by reactive oxygen species (ROS) that are produced by iron and would be expected to alter membrane fluidity and the activities of membrane enzymes [8,9,38,39]. The lipid profile of erythrocyte membranes in the patient group differed from that of the control group, as observed in Fig 2A. When the case group was separated according to age (Fig 2B), no significant difference was detected (p = 0.554) between patients over and under 50 years of age. However, when the case and control groups were both stratified by age group and compared, only the case and control groups aged over 50 years showed a significant difference in lipid profile (p < 0.005).

Patients suffering from iron overload presented a lower total membrane cholesterol content compared with that of the control group (p<0.001; case 0.729 μg/mL ± 0.083 vs control 1,331 μg/mL ± 0.125; Fig 2C). No significant difference was observed between patients when stratified by age (p = 0.241), as shown in Fig 2D. Comparative analysis between case and control groups that were stratified by age showed that the total cholesterol content was lower in the case groups relative to the control groups, with p = 0.049 for case and control groups containing individuals under 50 years of age and p = 0.002 for those aged over 50 years (data not shown).

### 3.4. Influence of iron overload on Na,K-ATPase activity

**Fig 3:**
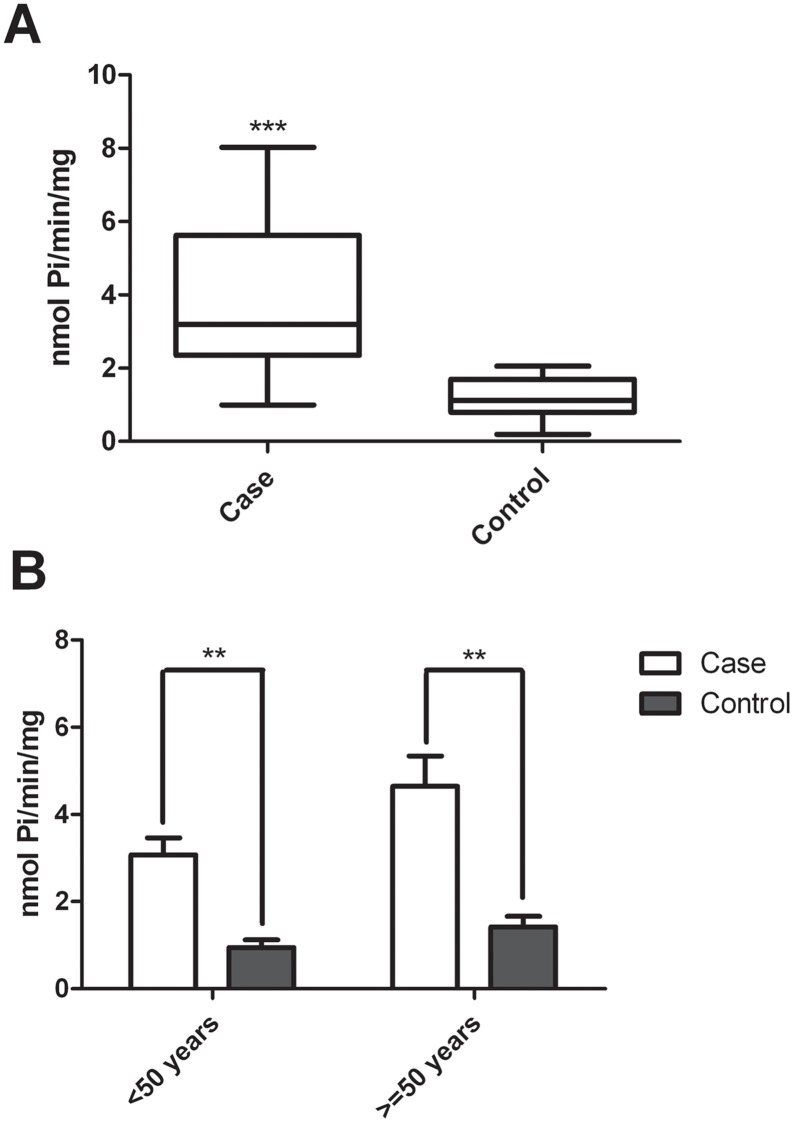
Influence of Fe^3+^ on the activity of Na,K-ATPase. (A) Activity of Na,K-ATPase in erythrocyte membranes of individuals with or without iron overload: n = 24 (case) and n = 12 (control), p<0.0001 (Mann Whitney). (B) Activity of Na,K-ATPase in erythrocyte membranes of subjects with or without iron overload, stratified by age group: n = 12 (<50 years), n = 06 (control <50 years), n = 12 (> = 50 years), n = 06 (Control > = 50 years). p = 0.002 (case x control <50 years), p = 0.005 (case x control > = 50 years), Student's t test. All experiments were performed in triplicates and the line in bars represented the mean and the error bar denotes SEM.

The lipid content of erythrocyte plasma membranes was significantly different between patients with iron overload and healthy volunteers. Therefore, we next assessed whether the activity of Na,K-ATPase also differed between the groups. Na,K-ATPase activity was significantly higher (by approximately 327%) in the erythrocyte membranes from the case group than in the control group (p<0.0001). Averages of 3.857 and 1.178 nmol Pi/min/mg were observed for the case and control groups, respectively (Fig 3A). When the groups were divided according to age, we also found an increase in Na,K-ATPase activity for patients with iron overload, as observed in Fig 3B.

### 3.5 Red blood cells treated with H_2_O_2_ and FeCl_3_

**Fig 4:**
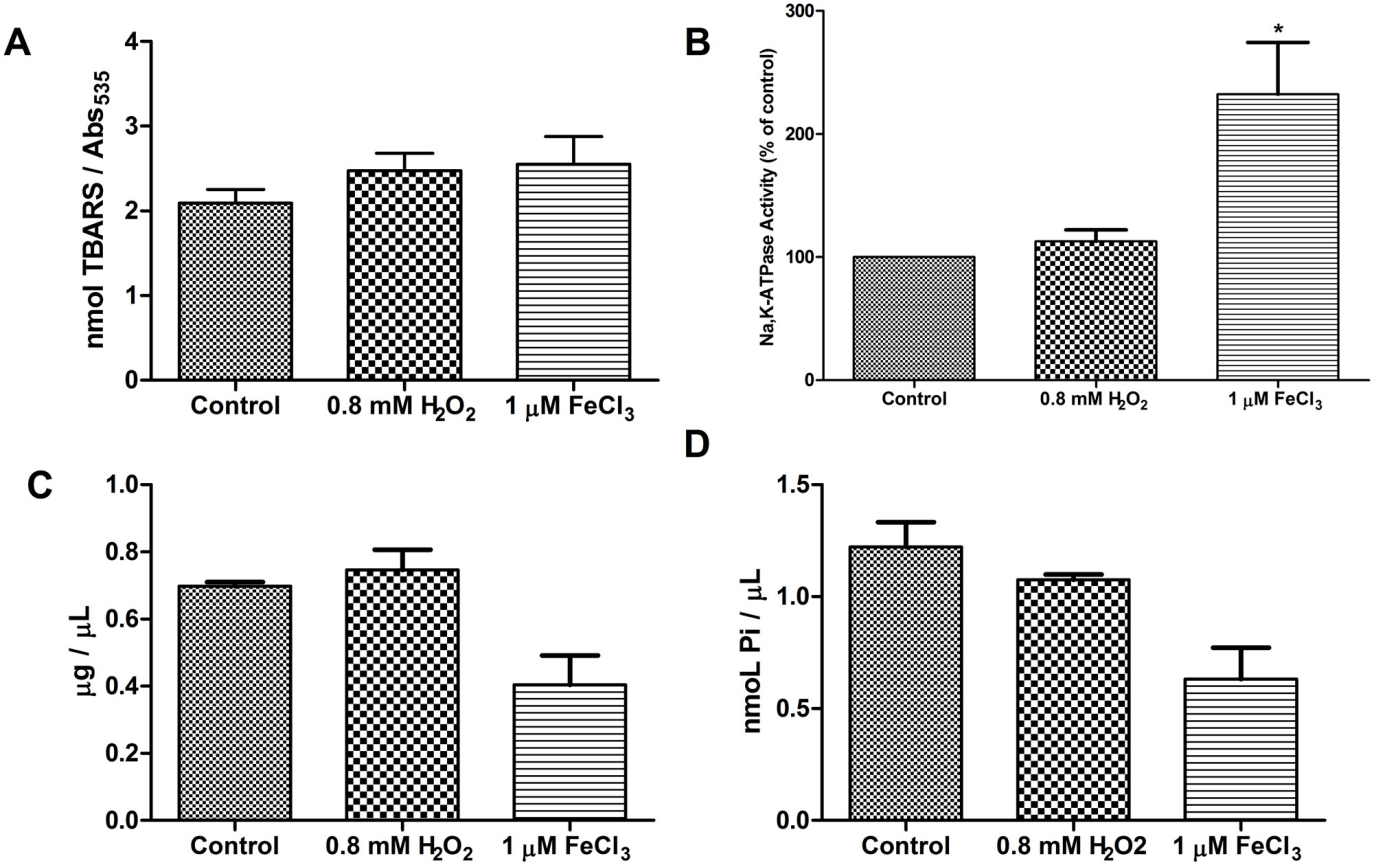
H_2_O_2_ and iron effect on red blood cell. Red blood cell from health donators were incubated with 0.8 mM H_2_O_2_ or 1 μM FeCl_3_ for 24h at 4°C. This sample was used to obtained: (A) plasma TBARS levels, (B) the Na,K-ATPase activity of membrane preparation and the total levels of (C) cholesterol and (D) phospholipids. All experiments were performed in triplicates and the line in bars represented the mean and the error bar denotes SEM, *p <0.05; results are significantly different from controls, as evaluated by ANOVA test.

To further examine the mechanism responsible for the changes in Na,K-ATPase and lipid modulation, we treated the red blood cells with 0.8 mM H_2_O_2_ and with 1 μM FeCl_3_. After treatment for 24 h, a total lipid extract and a membrane fraction were prepared. The treatment with 0.8 mM H_2_O_2_ did not cause any modification of the TBARS, the Na,K-ATPase activity or the lipid levels (Fig 4). However, 1 μM FeCl_3_ treatment induced similar modifications to those found in patients with iron overload; the Na,K-ATPase activity increased (Fig 4B), and the total cholesterol and phospholipids decreased (Fig 4C and 4D), though no difference in the TBARS level was observed (Fig 4A).

## Discussion

The Na,K-ATPase activity in erythrocytes presented a significant increase of approximately 327% in patients with iron overload compared to activity levels observed in the control group (p<0.0001). When analyzing this activity according to age, no significant difference was observed, even after a correlation of variables was implemented (p>0.05). However, when comparing the groups stratified by age, the activity of case patients remained high, at approximately 320%, in both age groups (p<0.01). The increase in the Na,K-ATPase activity may be related to several mechanisms, such as the modification of the microenvironment in which the enzyme is inserted, the action of transferrin, ROS activity, or the accumulation of intracellular Na^+^[8–9, 29, 38–39].

One of the first biochemical parameters that appear changed in patients with iron overload is transferrin saturation. Transferrin saturation reaches high values in these patients, generally above 80% [7]. Previous studies have demonstrated that transferrin is required for the activation of Na,K-ATPase at low concentrations of K^+^ and that this effect is mediated by enhanced activity and iron transport by ROS. In contrast, low K^+^ in the presence of transferrin caused an increased absorption of iron by MDCK cells. In this context, the increased activity of Na,K-ATPase appears to be a consequence of the intracellular accumulation of iron [29].

ROS are one of the products of oxidative reactions that participate in lipid peroxidation. ROS can also influence the stimulation of Na,K-ATPase. Zhou et al. demonstrated that low concentrations of K^+^ stimulate the intracellular activities of ROS and Na,K-ATPase activity in MDCK cells. The increase in Na,K-ATPase activity is mediated by ROS, which induces the gene expression of Na,K-ATPase, thereby increasing the number of enzymes and consequently its function[38].

Lipid peroxidation directly alters membrane fluidity, which is an important feature for maintaining the optimal functioning of erythrocytes. Membrane fluidity impacts the homeostatic control of erythrocytes, which in turn affects the passage of oxygen, water and ions, such as Na^+^, K^+^ and Ca^2+^, through the membrane, thus allowing equilibrium between the intracellular and extracellular media. Changes in the membrane dynamics and permeability caused by lipid peroxidation have also been reported[40–41]. Lipid peroxidation is one of the main sources of irreversible Ca^2+^ membrane permeability, which generates a series of changes in cell properties, including reduced membrane potential, increased permeability and loss of extracellular matrix components [42]. These environmental changes affect the kinetic parameters of enzymes such as Na,K-ATPase and modulate enzyme-substrate affinities [9]. In other cells, increased lysosomal fragility can lead to the release of proteolytic enzymes. Changes in the mitochondrial membrane can lead to cell death, and the stimulation of collagen deposition in the liver tissue can cause fibrosis and cirrhosis, which are often diagnosed in patients with untreated iron overload[43–44].

For the first time, we showed that the membrane lipid profile of red blood cells differs between patients with iron overload and healthy volunteers. Both the total phospholipid (p = 0.013) and cholesterol (p<0.001) contents were lower in the case group than in the control group. Changes in cholesterol and lipid fractions may influence ion transport, membrane fluidity and membrane permeability, thereby impairing the functional efficiency of the erythrocyte. Several modifications to the morphology of red blood cells have been demonstrated to result from iron effects[45–47]. Such effects lead to changes in the elasticity of membrane, thus hindering the passage of erythrocytes through narrow capillaries and affecting the lateral mobility of the Na,K-ATPase, both of which are critical to cell function[8, 48–50].

The Na,K-ATPase is confined to the plasma membrane of animals and is thus expected to be strongly affected by the diverse membrane content of cholesterol [51]. Indeed, every reaction step measured in the Na,K-ATPase reaction cycle was significantly affected by the concentration of cholesterol: the phosphoenzyme levels increased, the apparent ATP affinity increased, the cytoplasmic Na^+^ activation was inhibited at high Na^+^ concentrations, and the rate of phosphorylation and of spontaneous and K^+^-activated dephosphorylation increased[51–53]. Cholesterol molecules were recently shown to specifically bind to 3 different sites in the enzyme in high-resolution Na,K-ATPase crystal structures[54–55].

The internal K^+^ in red blood cells has been demonstrated to behave as a competitive inhibitor of internal Na^+^ binding and as an activator of maximal pump flux. Those researchers further noted that cholesterol depletion enhances each of these K+ effects. In the absence of internal K^+^, cholesterol depletion no longer has any effect on the enzyme [53].

However, only FeCl_3_ treatment caused similar modifications in red blood cells to those found in cells from iron overload patients. Direct treatment with 0.8 mM H_2_O_2_ for 24 h failed to induce any evident modification. Red blood cells have antioxidant defenses that catabolize H_2_O_2_ (such as catalase and the glutathione complex), but the hydroxyl radicals produced by the Fenton reaction due to changes in iron content cannot be directly inactivated and lead to increased cellular damage [56]. Moreover, this effect shows that iron modulation of Na,K-ATPase activity is not necessarily a result of increased expression of the pump on the cell membrane; instead, the increase may be caused by a direct interaction between iron and the enzyme pump or by the modulation of cholesterol and phospholipid content. Several papers have already demonstrated an effect of iron on Na,K-ATPase activity[57–58].

Approximately 70–76% of patients with iron overload have at least one of the recognized mutations in the HFE gene [5, 59–61]. In this study, 75% of patients in the case group had a mutant HFE genotype, although the proportion of mutant genotypes did not significantly differ between the case and control groups. In the control group, 42.8% of the participants had this mutated genotype, which suggests that the presence of the mutations in the HFE gene is not a determining factor for the occurrence of iron overload. Other factors have also been shown to induce iron overload[5, 62]. The most frequent mutant HFE genotype in case patients was H63D/WT (45.8% compared with 35.7% for the control group). However, in accordance with the literature results, patients with this genotype were not at a significantly increased risk of developing hemochromatosis and were unlikely to have symptoms of iron overload [5, 61–66]. In a recent study, H63D homozygosity was associated with an elevated mean ferritin level, but only 6.7% of the sixty patients studied had documented iron overload at follow-up [67].

Although those who are heterozygous for the C282Y mutation of the HFE gene do not usually express a hemochromatosis phenotype, 90–100% of typical hemochromatosis patients are homozygous for the C282Y mutation [68]. Here, we detected only 3 patients who were homozygous for this mutation, though the lack of correlation with iron overload may have been influenced by the small sample size.

The frequency of mutations in the HFE gene depends not only on the particular population but also on the criteria adopted for sample selection and the diagnosis of iron overload [69]. Because no studies on this subject have been previously published in Minas Gerais, a contextual evaluation of our results is not yet possible. It is likely that S65C mutations in the HFE gene were not identified in our study due to the low frequency of this mutation and the limited number of patients analyzed [70]. The frequency of the H63D mutation was higher for both the case (35.4%) and control subjects (17.9%), as expected [5, 59, 61–62, 66].

The main clinical manifestations reported by patients are consistent with those described in previous studies [63, 71–72], except for intense sweating, which was not previously reported. The human body has no specific mechanism for iron removal; consequently, it is possible that the body eliminates iron through sweat [73]. Thus, this pattern of increased sweating of patients with iron overload appears to be an adaptation by the body to increased iron content. Stratifying both groups by age revealed that skin hyperpigmentation presents more frequently in patients under 50 years (p = 0.012). Hyperpigmentation of the skin is likely due to iron-induced increases in the melanin or iron deposits around sudoriparous glands [74]. The differences observed between the age groups are likely the result of increased levels of iron accumulation in older patients because the iron overload likely occurs for a longer period than experienced by younger patients. Importantly, the absence of differences found between the patients and control subjects may not be a reflection of disease effects but rather may be due to the small sample sizes, as only 2 data sources were used in some strata (Table 1).

## Conclusions

In erythrocytes, iron overload can compromise functional efficiency, decrease membrane phospholipid and cholesterol content, and increase lipid peroxidation and Na,K-ATPase activity. These effects are specifically modulated by the increase in iron content and are not simply a result of the consequent increase in intracellular ROS.
